# Effect of nitrogen on grain growth and formability of Ti-stabilized ferritic stainless steels

**DOI:** 10.1038/s41598-019-42879-3

**Published:** 2019-04-24

**Authors:** Mun Hyung Lee, Rosa Kim, Joo Hyun Park

**Affiliations:** Department of Materials Engineering, Hanyang University, Ansan, 15588 Korea

**Keywords:** Structural materials, Metals and alloys

## Abstract

The relationship between the grain size of as-cast and cold rolled 16%Cr ferritic stainless steel and the surface roughness defect, called *ridging* during forming was investigated. The ridging height corresponded to the grain size of the as-cast sample. The nitrogen content of 140 ppm yielded the minimum grain size and the minimum ridging height observed, whereas the nitrogen content of 50 ppm yielded the maximum grain size and the maximum ridging height observed. Ridging results from different plastic anisotropies of band structure composed of colonies. Through the EBSD analysis, the texture of mixed colonies composed of ND//{112} and ND//{331} in the 50 ppm nitrogen steel underwent more severe ridging than the randomly texture in the 140 ppm nitrogen steel sample. Therefore, an effective means to reduce the ridging of ferritic stainless steel during the forming process is to form a random texture by enhancing the formation of fine equiaxed grain during the casting process. During equal holding times at 1200 °C, the 80 ppm nitrogen sample was definitely coarsened, whereas the 200 ppm nitrogen sample underwent slower grain growth. Zener pinning force, which is proportional to the number of TiN particles on grain boundaries, was relatively strong in samples of 200 ppm nitrogen content, corresponding to slower grain growth. Although the Zener pinning force great affected with increasing nitrogen content, there may not affect the trend of initial cast grain size to be changed as much during annealing.

## Introduction

Ferritic stainless steels (FSSs) are widely used in automobile manufacturing and in various other fields because of their low thermal expansion and high resistance to corrosion. However, their limited formability due to ‘ridging’ defects must be resolved to expand their use. Ridging is typically observed on the surface of an FSS sheet after cold forming or deep drawing, degrading the surface quality of the sheet. “Many researchers have observed” that ridging arises from differences of plastic strain anisotropies between the matrix and colonies^1–14^.

Viana *et al*.^11^ studied the ridging behavior of three kinds of FSS and reported that ridging was largely dependent on the plastic behavior of the colonies. They claimed that the ridging arose from the different plastic behaviors of {111} 〈uvw〉 and {001} 〈uvw〉 grain colonies in the sheet. Shin *et al*.^5^ studied the effect of texture on ridging of 430 (16%Cr) and 409L (11%Cr) grade FSSs and tested ridging models quantitatively using a simulation tool. The 409L showed more severe ridging than 430 steel, and colonies found in 409L steel specimens well represented the tendency of ridging. Their simulation results showed that low plastic strain ratios of colonies gave rise to ridging.

Various methods have been proposed to improve ridging. Huh and Engler studied the effect of intermediate annealing on ridging in 17%Cr FSS^3^, reporting that a sheet that underwent intermediate annealing showed much less ridging than an ordinarily rolled sheet. During intermediate annealing, recrystallization gave rise to the more desirable γ-fiber texture, which was reflected by a greater R-value and led to improved formability of the sheet. Huh *et al*.^7^ also reduced the ridging phenomenon by means of cross rolling. Modification of the initial texture and microstructure by means of 45° ND cross rolling resulted in improved macro- and microscale texture in the finally recrystallized sheets. These modifications led to enhanced planar anisotropy and less ridging. Tsuchiyama *et al*.^8^ studied the production of ridging-free FSS through the recrystallization of lath martensitic structure, by which the flat surfaces after a 20% tensile test were formed with crystallographically isotropic or near isotropic ferritic structures with no colonies. The lath martensitic structure was originally isotropic due to the multi-valent transformation mechanism.

Alternatively, to eliminate the colony structure that causes the ridging phenomenon, many researchers have tried to form fine equiaxed grains during solidification process^15–23^. Park *et al*.^15–18^ studied the grain refinement using inoculants. The grain size of the as-cast structure decreased with increasing content of Ti in an Al-Ti deoxidized 16%Cr FSS, and single TiN as well as MgAl_2_O_4_-TiN complex inclusions were formed in steels with fine equiaxed grains. Takeuchi *et al*.^19^ studied the effect of electromagnetic stirring on cast structure of 430 steels. The equiaxed crystal zone was increased with increasing stirring intensity. We previously varied the nitrogen content as a means to refine the cast structure, specifically by controlling the number density of TiN particles at a specific nitrogen content during melting and casting of 16%Cr FSS^23^. It was confirmed that fine equiaxed structure was promoted as the number of TiN particles per unit area (or volume) in the melt increased.

Even though there are many studies about refinement of the solidification structure in order to reduce ridging phenomenon, the effect of grain growth by pinning particles on the degree of ridging is not certain. Therefore, the aim of the present study is to evaluate the influence of the equiaxed grain formation in the cast samples and that of grain growth on the degree of ridging of final products, and to identify which is the more dominant factor affecting the ridging height. Hence, we analyzed the Zener pinning effect of TiN particles on grain boundaries and discussed its influence on grain growth during annealing at 1200 °C.

## Materials and Methods

### Materials preparation and measurement of ridging height

**Figure 1:**
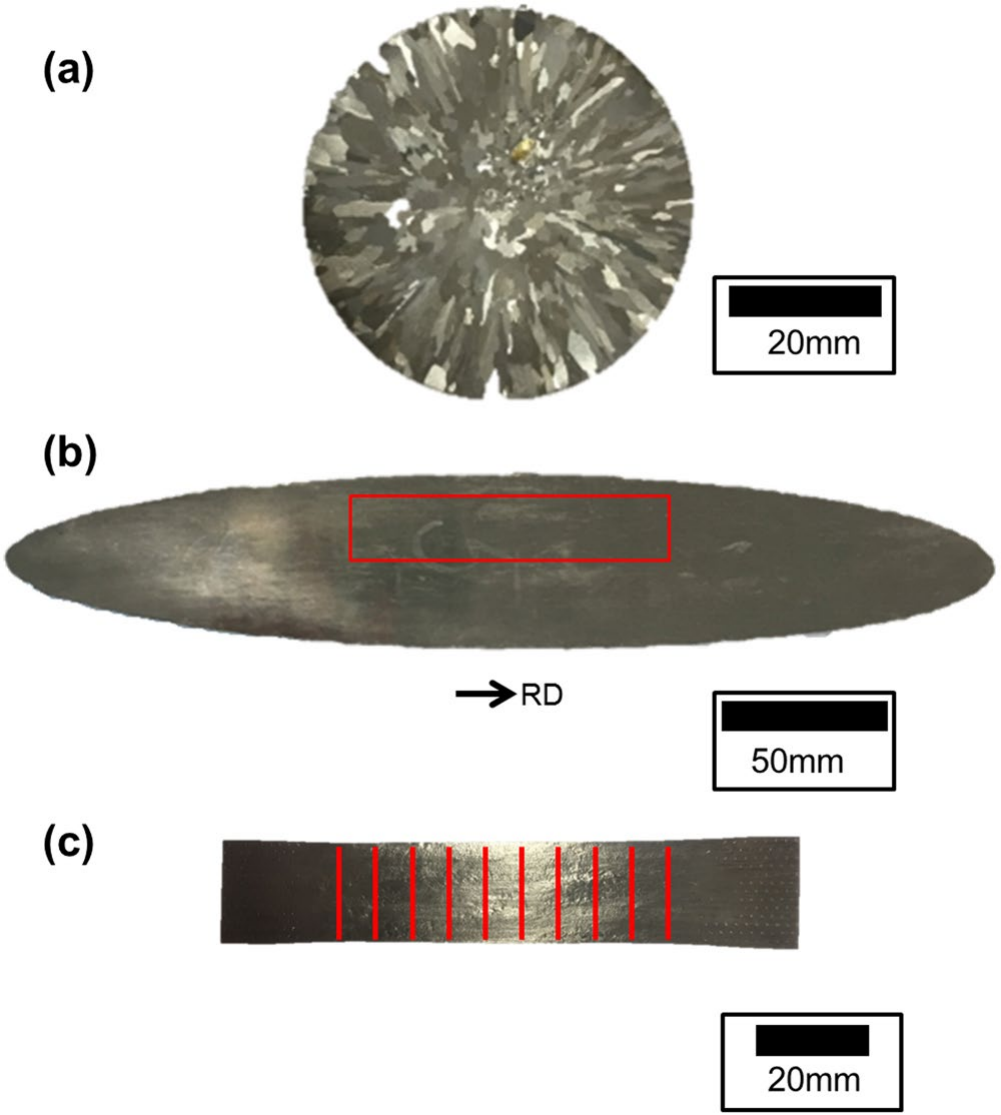
Sample preparation and measurement of ridging height. (**a**) as-cast ingot, (**b**) cold rolled sheet, and (**c**) 15% deformed sample.

In our previous study^23^, a Fe-16Cr-0.3Mn-0.3Si-0.2Ti-0.03Al-0.004 C (wt%) steel was melted and supplemented with nitrogen at 1550 °C using an induction furnace. The furnace was then turned off, and the melt was solidified at a cooling rate of about −50 °C/min to yield a cylindrical cast ingot. The chemical compositions of solidified ingots are listed in Table 1. To measure the ridging height of the sheet, samples of the cylindrical cast ingot (diameter: 50 mm, height: 10 mm), which included grains of various sizes, were cold rolled to 1.0 mm thickness. The cold rolled sheets with 90% reduction rate were cut to 100 mm in length and 20 mm in width, which were then annealed at 1050 °C for 120 sec, cooled, and surface pickled. The resulting samples were deformed to 15% tensile elongation along the rolling direction. The shape of as-cast ingot, cold rolled sheet and 15% deformed sample with 10 positions for ridging height measurement are illustrated in Fig. 1. The surface roughness profile of the sheets was measured using an alpha step (ET200; Kosaka Ltd., Japan). Ridging heights were obtained as average values of 10 surface measurements performed in different places on each sample.

**Table 1:** Chemical composition of materials (wt%).

Sample ID	Titanium	Aluminum	Oxygen	Nitrogen
50N	0.16	0.010	0.0027	0.0050
80N	0.17	0.013	0.0025	0.0080
140N	0.14	0.011	0.0034	0.0140
200N	0.15	0.009	0.0030	0.0200
260N	0.13	0.011	0.0035	0.0260

^*^[Cr] = 16.0 wt%, [Mn] = 0.3 wt%, [Si] = 0.3 wt%, [C] = 0.004 wt%.

### Texture analysis

To prepare samples for texture analysis, annealed sheets were polished with 2000 SiC paper and then electrically polished under 43 V in a solution of 40 mL perchloric acid and 1 L ethanol. Local texture were measured by field emission scanning electron microscopy paired with energy dispersive X-ray spectroscopy (FESEM-EDS; MIRA3; TESCAN Ltd., Czech) and electron backscatter diffraction analysis (EBSD). Crystallographic orientation measurements were performed on the ND planes of the sheets.

### Characterization of grain size and TiN precipitates after isothermal heat treatment

Specimens of 10 mm × 10 mm were cut from the centers of the ingot samples with nitrogen contents of 80, 140 and 200 ppm. The specimens were heated at 1200 °C and held at that temperature for 2, 10, and 60 min, followed by water quenching to room temperature. The specimens were polished and etched (in a mixture of 2 g picric acid, 90 mL ethanol, and 10 mL HCl), and then their grains were observed at × 50 magnification using optical microscopy (OM; GX41, Olympus, Essex, UK); the total observation area for each specimen was 40 mm^2^. The average grain size in each observation area was measured using automatic image analysis computer software. Density and size of TiN particles was also observed using optical microscopy and transmission electron microscope (TEM; JEM-2100F, JEOL, Japan). The TEM sample was prepared by carbon replica technique.

## Results and Discussion

### Effect of As-cast grain size on ridging height

**Figure 2:**
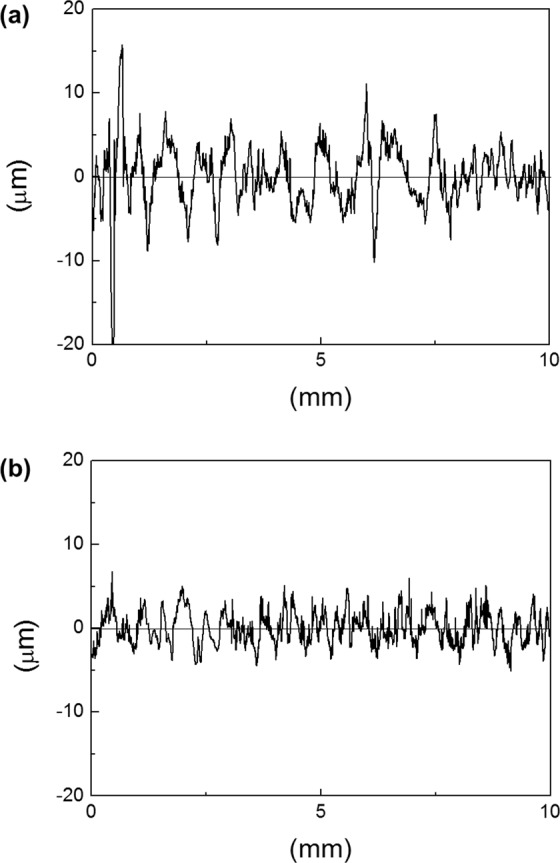
Surface profiles of (**a**) 50 ppm nitrogen and (**b**) 140 ppm nitrogen sheets deformed to 15% elongation in rolling direction.

**Figure 3:**
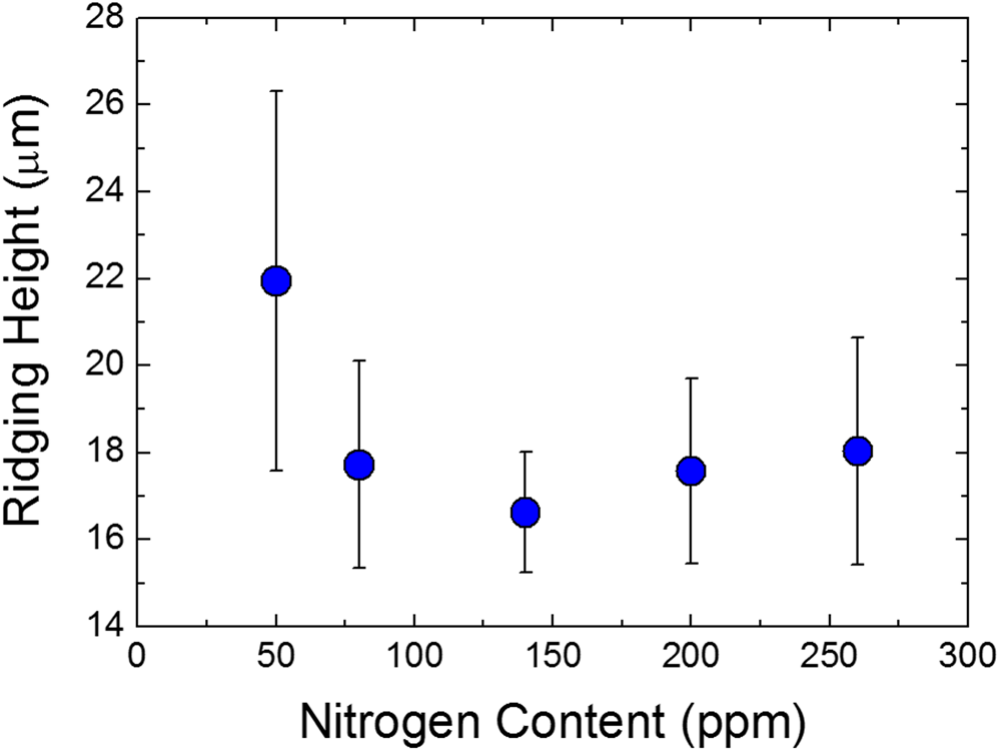
Ridging height as a function of nitrogen content.

**Figure 4:**
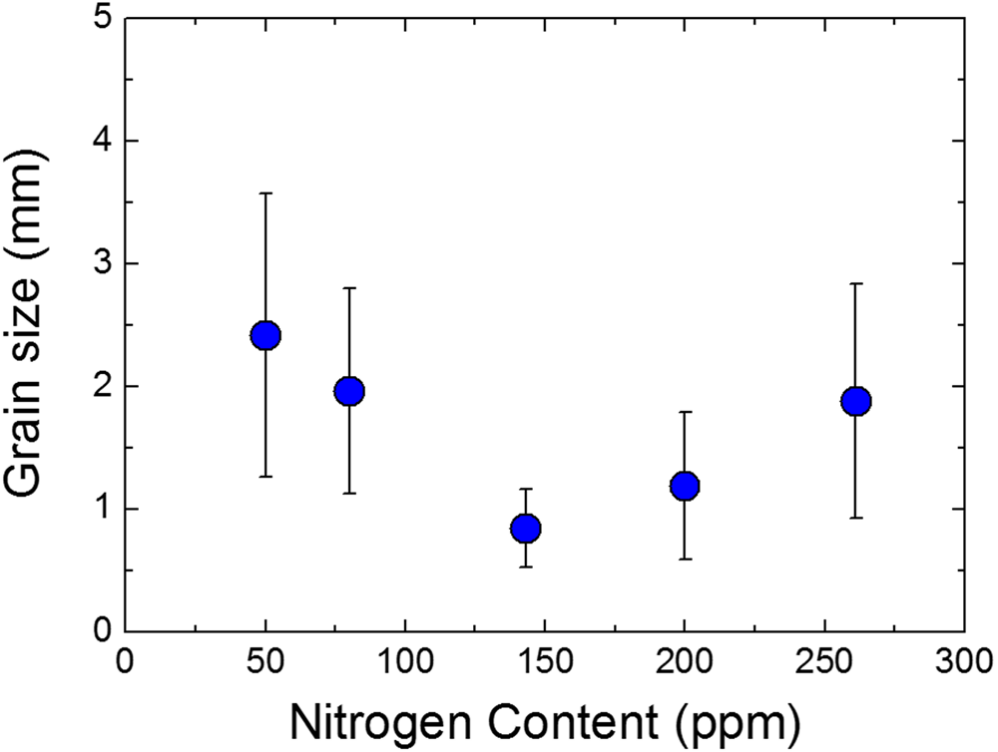
Average grain size of as-cast sample as a function of nitrogen content.

Surface profiles of cold rolled sheets after 15% tensile deformation are shown in Fig. 2. The sheet with a nitrogen content of 50 ppm yielded the poorest surface quality (Fig. 2a), whereas the sheet with higher nitrogen content of 140 ppm yielded the smoothest surface (Fig. 2b). Ridging height is defined as the height difference between the valley and the peak of a surface profile; ridging heights averaged from 10 measurements are plotted versus nitrogen content in Fig. 3. The ridging height was lowest at the nitrogen content of 140 ppm and highest at the nitrogen content of 50 ppm. The trend in ridging height generally matched the trend in grain size of as cast samples reported in our previous study^23^; the minimum grain size and minimum ridging height were both observed at the nitrogen content of 140 ppm, and the maximum grain size and maximum ridging height were both observed at the nitrogen content of 50 ppm (Fig. 4).

### Effect of texture on ridging height

**Figure 5:**
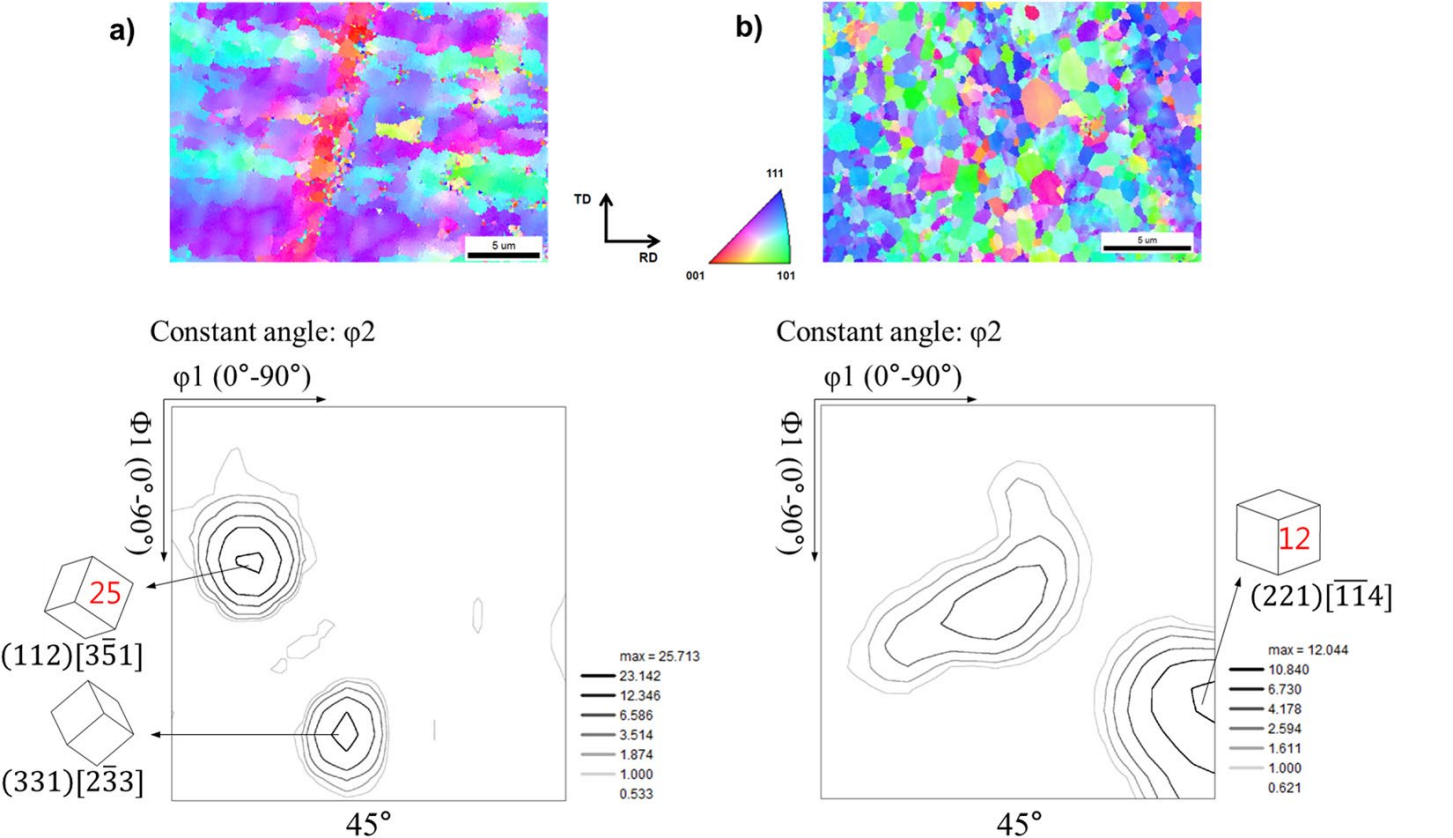
EBSD inverse pole figure maps from ND plane and orientation distribution function (ϕ2 = 45°) of (**a**) 50 ppm N and (**b**) 140 ppm N containing sheets after cold rolling and annealing.

Figure 5 shows inverse pole figure (IPF) maps indicating the crystallographic axis of each point parallel to the normal direction (ND) in specimens from sheets of nitrogen content 50 ppm (Fig. 5a) and 140 ppm (Fig. 5b). At the bottom of each figure, corresponding ODF sections, at φ2 = 45° (Bunge notation) are attached together. In IPF map, red color code denotes the {100}//ND cube orientation, and blue denotes the {111}//ND γ-fiber orientation. Generally, the R-value depends on the relationship between the grain orientation and the deformation direction^5^. The sheet of nitrogen content 50 ppm mainly comprised orientation colonies close to {112}//ND and {331}//ND. The ODF maximal value clearly indicates very sharp texture in those directions. Ma *et al*.^10^ conducted a simulation to predict ridging height. They inserted specifically oriented colonies into the matrix and simulated the ridging height. The colonies and the matrix with different texture showed plastic anisotropies, resulting in large difference in calculated ridging heights. It is deduced that the band structure comprised of large colonies formed by grains with similar orientations, led to plastic anisotropy during the tensile test and thus caused ridging. On the other hand, the sheet of nitrogen content 140 ppm comprised relatively weak texture, with maximal value of 12 near (221) $[\overline{11}4]$. The increase of randomness in the crystallographic orientation of grains would have helped this structure receive almost equal strain in all grains during the tensile test and thus minimize the ridging.

In steel sheets that showed severe ridging, the initial solidification structure was usually coarse and columnar, whereas the initial solidification structure of steel sheets represented minimum ridging was fine and equiaxed. The columnar structure develops long grain colonies during cold rolling and survives even after annealing^5^. Therefore, one way to reduce the ridging phenomenon of ferritic stainless steel during the forming process is to form a random texture by enhancing the formation of fine equiaxed grain during the solidification process.

### Relationship between number density of TiN particles and grain growth behavior

**Figure 6:**
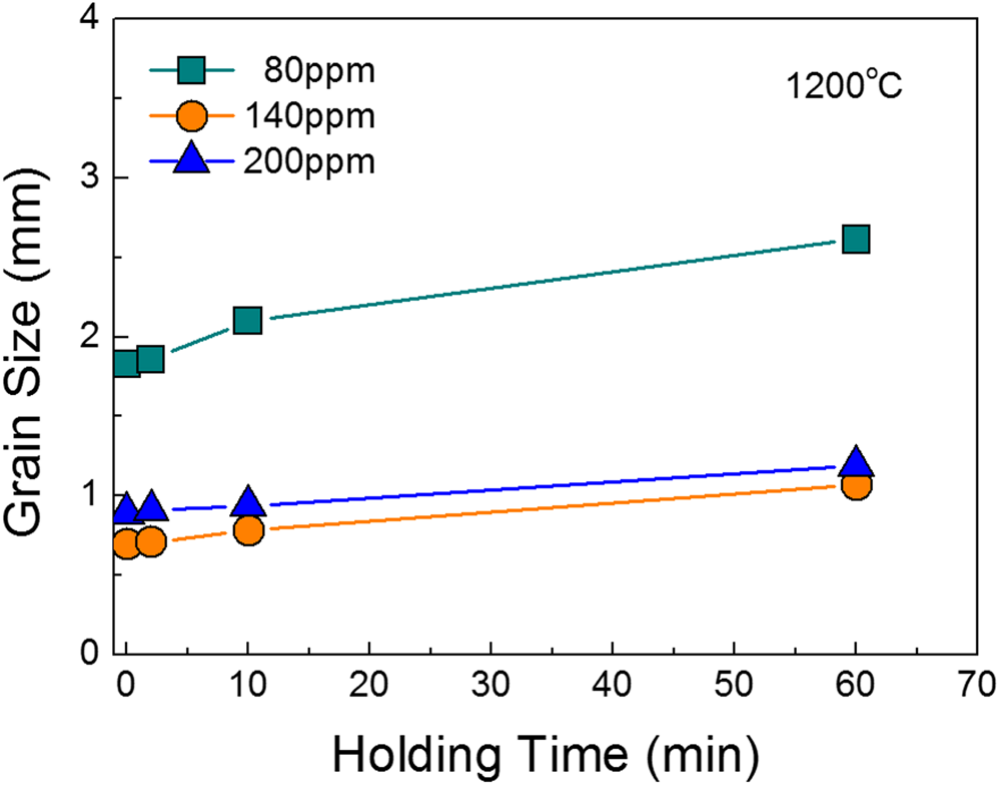
Grain size of annealed samples with various nitrogen contents as a function of holding time at 1200 °C.

The grain size of heat treated samples with different nitrogen contents is plotted in Fig. 6 against holding time at 1200 °C. During the overall holding time of 60 min, the grain definitely coarsened in the sample with a nitrogen content of 80 ppm, while the 200 ppm sample underwent slower grain growth. This can be explained by the difference in fraction of TiN particles observed on grain boundaries. The fraction of particles on grain boundary was calculated by the number of TiN particles on grain boundary divided by the total number of TiN particles in the observation areas^24^. The fraction of particles was increased with increasing nitrogen content as listed in Table 2.

**Table 2:** Fraction of particles on grain boundary (%); The number of TiN particles on grain boundary divided by the total number of TiN particles.

Holding time at 1200 °C	80 ppm N	140 ppm N	200 ppm N
0 min	2.8	50.0	62.8
2 min	2.0	42.3	66.6
10 min	1.7	36.8	60.0
60 min	2.2	29.4	43.4

**Figure 7:**
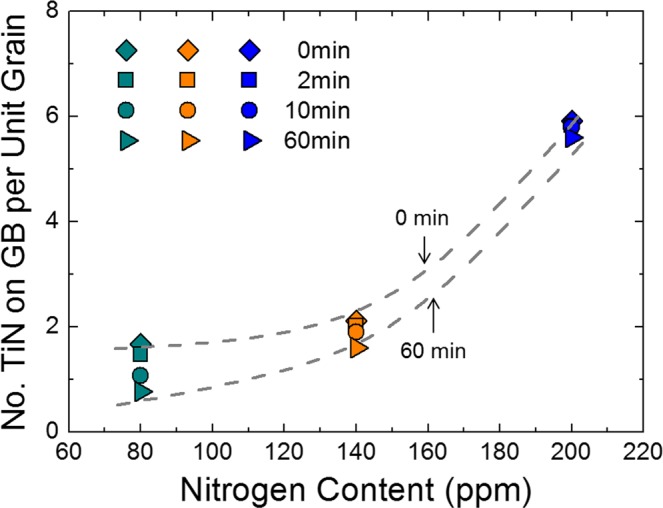
Number of TiN particles on grain boundary per unit grain as a function of nitrogen content.

**Figure 8:**
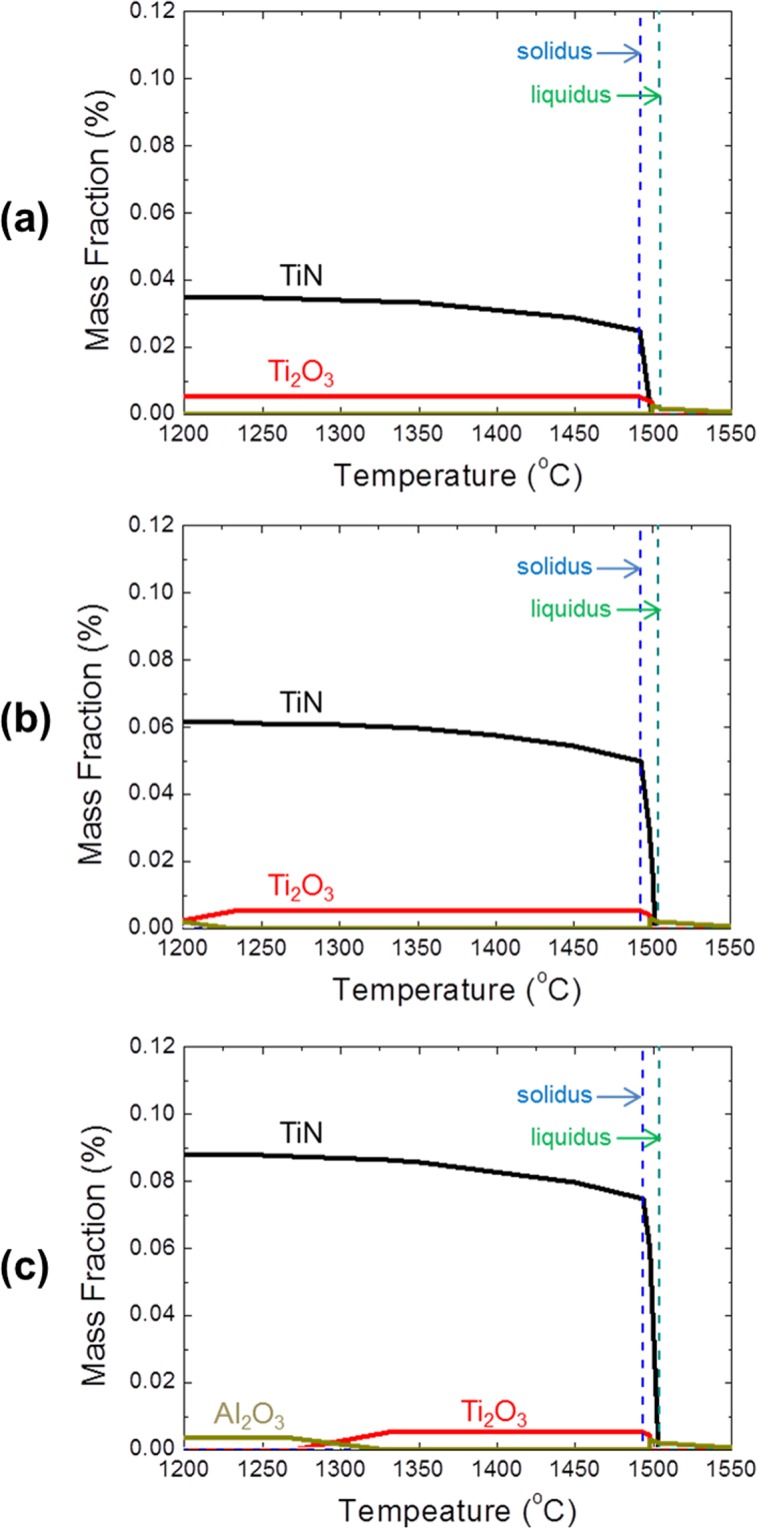
Simulated equilibrium cooling curve for Fe-16%Cr-0.3%Mn-0.3%Si-0.2%Ti-0.03%Al-0.004%C-0.0001%Mg-0.002%O-N system using FactSage^TM^ with nitrogen content of (**a**) 80, (**b**) 140 and (**c**) 200 ppm.

If a greater number of grains exist in a given area, there is a possibility that more TiN particles exist on their boundaries. Hence, to accurately count the number density of TiN particles on grain boundaries, the number of TiN particles on grain boundaries was divided by the total number of grains in the observation areas; the results are plotted against nitrogen content in Fig. 7, showing that the number of TiN particles on grain boundaries per single grain also increased with increasing nitrogen content. Figure 8 shows the equilibrium cooling curves for the Fe-16Cr-0.3Mn-0.3Si-0.2Ti-0.03Al-0.004C-0.0001Mg-0.002O-N (wt%) system calculated using FactSage^TM^ software (version 7.0) for the nitrogen contents of 80, 140, and 200 ppm^25^. The thermodynamic calculations confirmed that the content of TiN particles increased with increasing nitrogen content under equilibrium conditions.

TiN particles present on grain boundaries play a role in the retardation of grain growth. The retardation force by these particles is called the Zener pinning force and can be expressed as follows^26^;

1 $$Z=\frac{3\sigma V{f}_{v}}{r},$$

where *σ* is the grain boundary energy (=6 × 10^−7^ J/mm^2^)^27^, *V* is the molar volume of Fe-16%Cr steel (=7.04 × 10^3^ mm^3^/mol), *f*_*v*_ is the particle volume fraction, and *r* is the particle radius. The restraining force due to these particles is directly proportional to their volume fraction and inversely proportional to their size. The *f*_*v*_ can be estimated from the spatial size distribution of TiN particles as expressed in Eqs (2) and (3)^28^.

2 $${f}_{v}=(\frac{{\pi }^{3}}{24})\cdot {\overline{d}}^{2}\cdot {N}_{a}$$

3 $$\frac{{\rm{1}}}{\overline{d}}=\frac{{\rm{1}}}{n}\cdot \sum \frac{{\rm{1}}}{{d}_{i}}$$

where *N*_*a*_ is the number of inclusions per unit area in the specimen, $\overline{d}$ is the harmonic mean of the TiN particle size, and *d*_*i*_ is the cross-sectional particle diameter.

**Figure 9:**
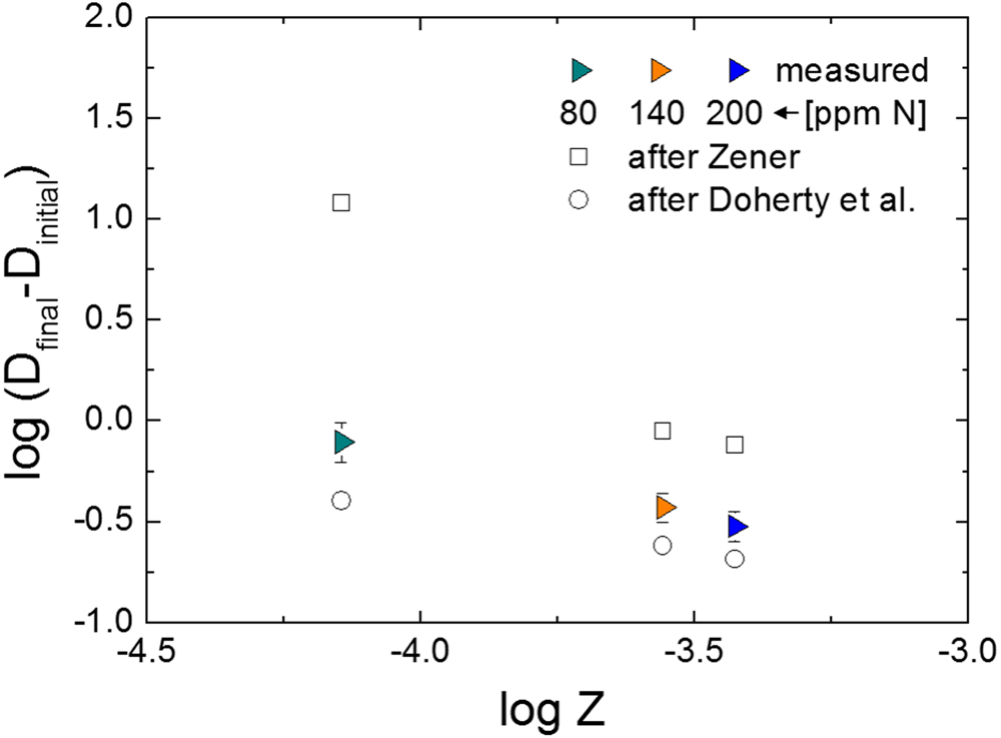
Relationship between Zener pinning force and the difference in grain size between before and after 60 min heat treatment at 1200 °C.

The relationship between Zener pinning force and the difference of grain size between before and after 60 min heat treatment is represented in Fig. 9. The Zener pinning force increased with increasing nitrogen content, corresponding to increased force suppressing the grain growth. Sasaki *et al*.^29^ studied the effects of titanium addition on the microstructure of S45C steel, finding that increments of Ti addition decreased the grain size. They explained that the grain size decreased with increasing Zener force. Ohta *et al*.^27^ also studied the inhibition of austenite grain growth by TiN particles, reporting that the grain size decreased with increasing Zener force when the TiN particles were uniformly dispersed. Janis *et al*.^30^ studied the effect of particles on the migration of grain boundaries in a Fe–20%Cr alloy, reporting that grain size decreased with increasing particle number. The change in the pinning effect according to particle number was explained using the ratio of the perimeter to the area of grain, P_GB_/A_G_. Larger values of P_GB_/A_G_, which correspond to large grain boundary curvatures, corresponded to smaller grains.

Zener reported that grain size D would reach a limiting value depending on the particle radius (*r*) and particle volume fraction (*f*_*v*_)^26^. Since then, many researchers reported the relationship between D, *r* and *f*_*v*_^31–34^. The general form of the equation is as follows;

4 $$D=\beta \cdot \frac{r}{{f}_{v}^{m}}$$

where β is pinning factor and *m* is volume fraction exponent. In Fig. 9, results of theoretical value were plotted as open shape. Open square is from the Zener’s theory, which is expressed by the following equation^26^;

5 $$D=\frac{4r}{3f}$$

However, Zener’s theory assumed that the grain has completely isotropic circle and particles are dispersed randomly. Open circle in Fig. 9 is from the Doherty *et al*.’s theory, which is expressed by the following equation^31^;

6 $$D=\frac{2d}{{({\rm{\beta }}{\rm{\Phi }}f)}^{1/2}}$$

Doherty *et al*.’s theory takes into consideration the fraction of particles on grain boundary (**Φ**) and radius of curvature of grain boundary (β). Through the computational simulation, **Φ** = 0.5 and β = 2 were obtained, indicating that half of particles are located on grain boundary and the radius of curvature of grain boundary is twice the radius of the average grain. Comparing two equations, the Doherty *et al*.’s equation well predicted the present experimental results as shown in Fig. 9. As shown in Table 1, approx. half of particles, i.e., 39.6(±8.7)% and 58.2(±9.2)% are located on grain boundary in 140 ppm N and 200 ppm N sample, respectively. Therefore, it is suggested that half of particles located on grain boundary possibly affected the retardation of grain growth.

**Figure 10:**
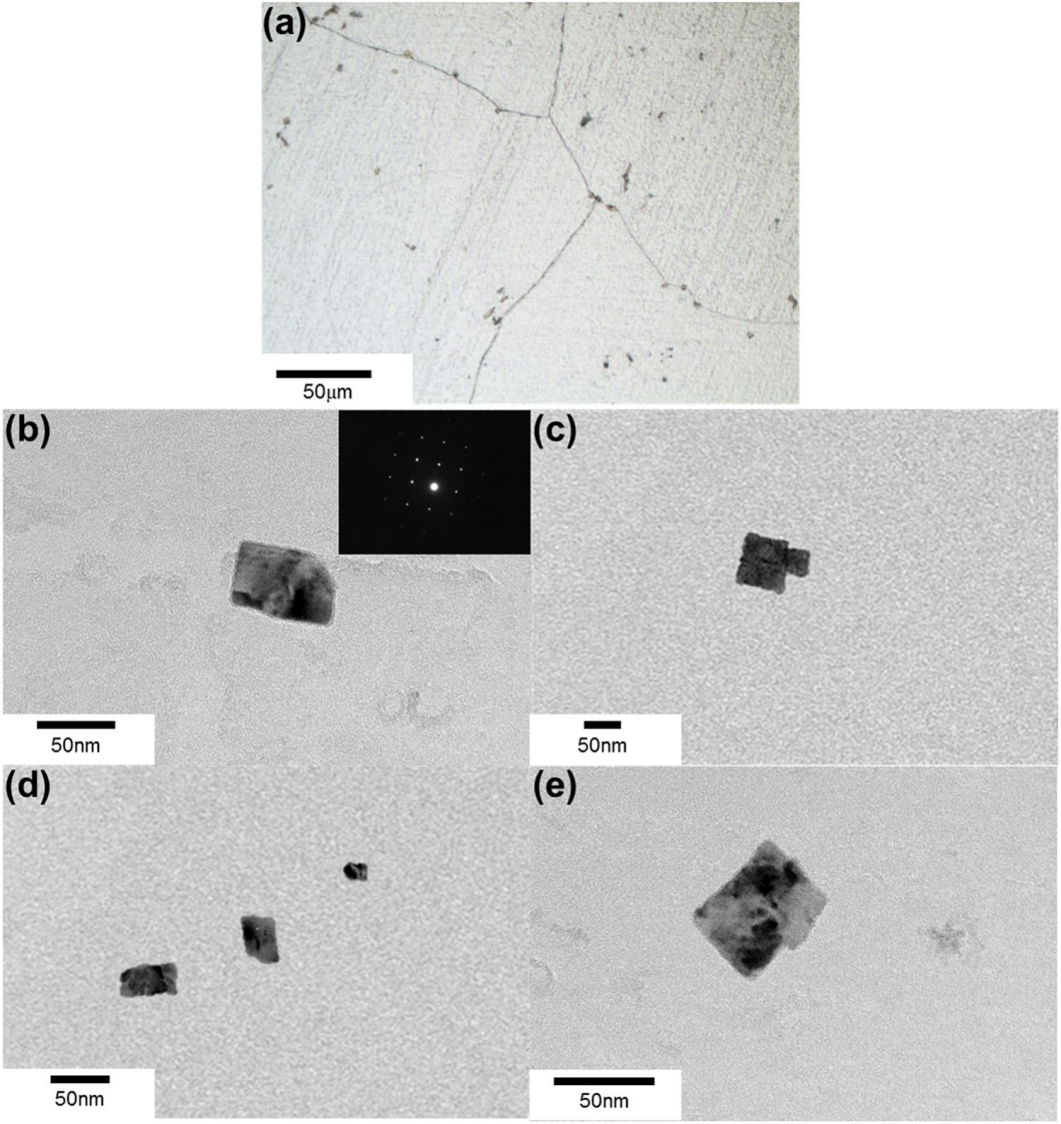
TiN particles after 10 min heat treatment. (**a**) TiN particles observed by optical microscopy; (**b**–**e**) TiN particles observed by TEM.

**Figure 11:**
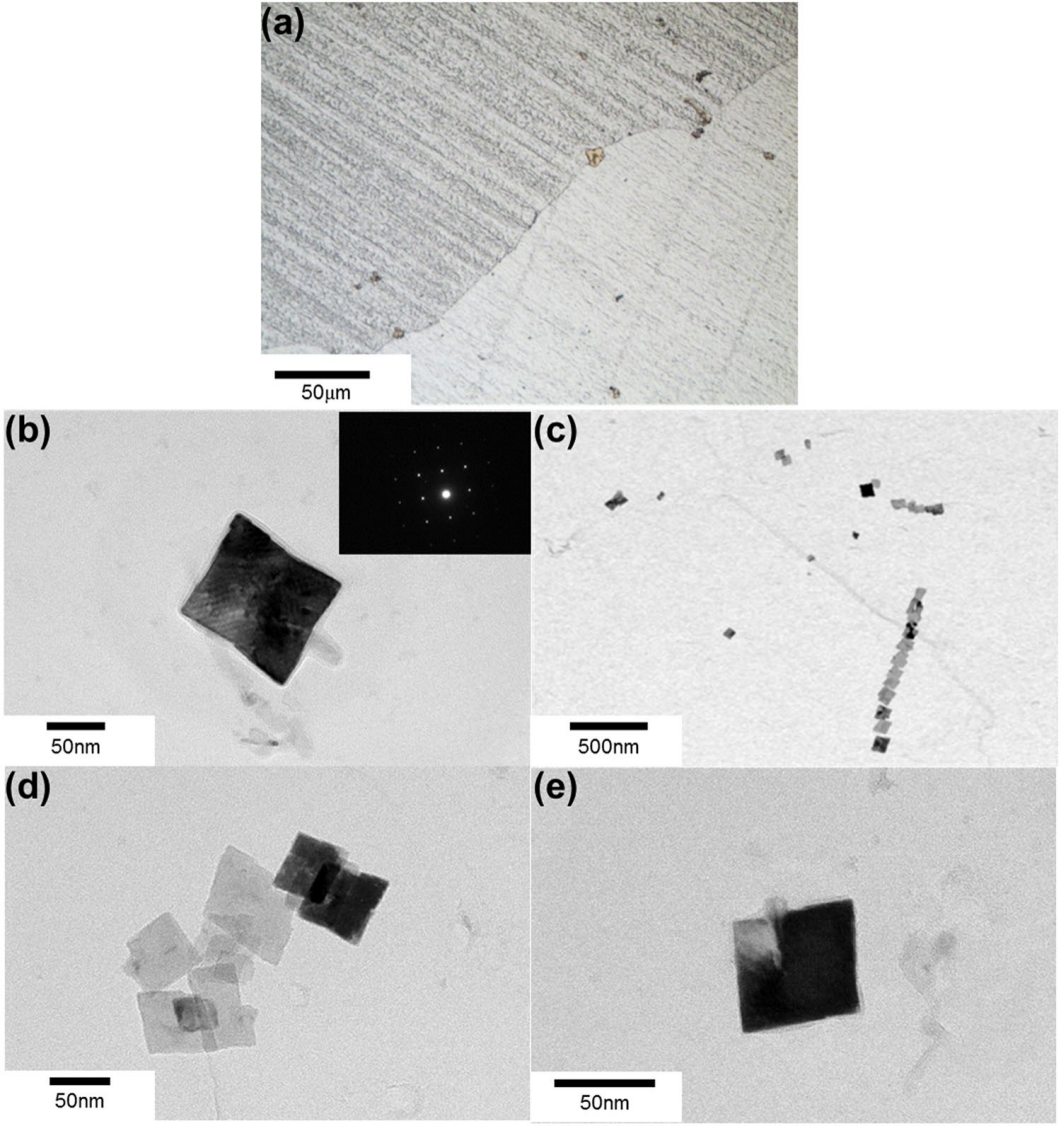
TiN particles after 60 min heat treatment. (**a**) TiN particles observed by optical microscopy; (**b**–**e**) TiN particles observed by TEM.

In Fig. 6, grain size increased with increasing holding time, irrespective of the nitrogen content. This originated from the fact that the size of TiN increased with increasing holding time, i.e., Zener force, inversely proportional to the particle size, became weak. The TiN particles found in 10 min and 60 min heat treated sample (80 ppm N) are shown in Figs 10 and 11, respectively. Figures 10a and 11a represent the TiN particles observed by optical microscopy. Figures 10b–e and 11b-e exhibit the cuboidal TiN particles observed by TEM and through SAD pattern and EDS analysis. The average TiN particles size was about 50 nm and 100 nm in 10 min and 60 min heat treated sample, respectively.

**Figure 12:**
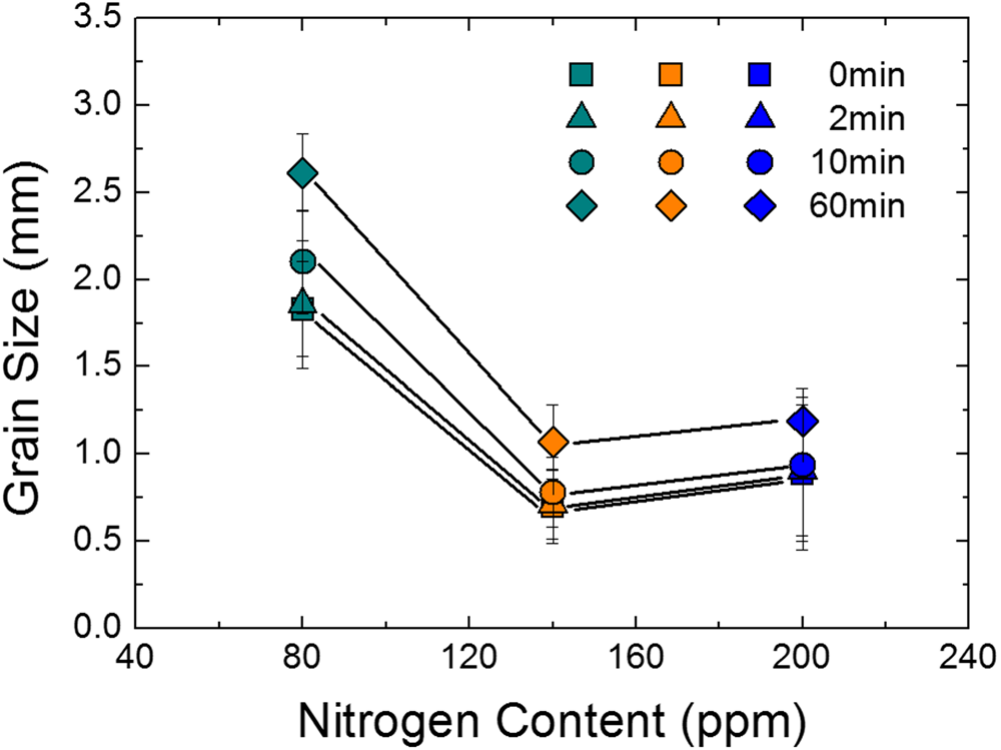
Changes in grain size of annealed samples according to holding time at 1200 °C as a function of nitrogen content.

From the above results, although the Zener pinning force affected the grain growth during heat treatment, it did not reverse the original trend of as-cast grain size as shown in Fig. 12. Therefore, the formation of fine equiaxed grain during solidification is more effective to diminish ridging height than retardation force of grain growth during annealing.

## Conclusions

We investigated the influence of the equiaxed grain formation in the cast samples on the degree of ridging. Also, we quantitatively characterized TiN particles on grain boundaries and discussed its influence on grain growth during annealing at 1200 °C to clarify which is the more dominant factor affecting the ridging phenomenon. The major findings of the present study are as follows.

- The ridging height corresponded to the grain size of the solidified sample. The nitrogen content of 140 ppm yielded the minimum grain size and the minimum ridging height observed, whereas the nitrogen content of 50 ppm yielded the maximum grain size and the maximum ridging height observed.
- Ridging results from different plastic anisotropies of band structure composed of colonies. The 50 ppm nitrogen sample with mixed colonies composed of ND//{112} and ND//{331} texture underwent more severe ridging than the 140 ppm nitrogen sample which has weaker texture. Therefore, an effective means to reduce the ridging of ferritic stainless steel during the forming process is alleviated texture by enhancing the formation of fine equiaxed grain during the solidification process.
- During equal holding times at 1473 K (1200 °C), the 80 ppm nitrogen sample was definitely coarsened, whereas the 200 ppm nitrogen sample underwent slower grain growth. The Zener pinning force, which is directly proportional to the number of TiN particles on grain boundaries, was relatively strong in samples with a 200 ppm nitrogen content and thus retarded the grain growth.
- Although the Zener pinning force affected the grain growth during annealing, it did not reverse the original trend of as-cast grain size, indicating that the formation of fine equiaxed grain during solidification is more effective to diminish ridging height than retardation force of grain growth during annealing.

## Data Availability

The datasets generated during the current study are available from the corresponding author on reasonable request.
